# Structural Evaluation and Electrophysiological Effects of Some Kynurenic Acid Analogs

**DOI:** 10.3390/molecules24193502

**Published:** 2019-09-26

**Authors:** Evelin Fehér, István Szatmári, Tamás Dudás, Anna Zalatnai, Tamás Farkas, Bálint Lőrinczi, Ferenc Fülöp, László Vécsei, József Toldi

**Affiliations:** 1 Department of Neurology, Interdisciplinary Excellence Centre, Albert Szent-Györgyi Clinical Center, Faculty of Medicine, University of Szeged, Semmelweis u. 6, H-6725 Szeged, Hungary; feher.evelin23@gmail.com (E.F.); vecsei.laszlo@med.u-szeged.hu (L.V.); 2 Department of Physiology, Anatomy and Neuroscience, University of Szeged, Közép fasor 52, H-6726 Szeged, Hungary; dudi.14t@gmail.com (T.D.); zalatnaianna@gmail.com (A.Z.); toldi@bio.u-szeged.hu (J.T.); 3 Institute of Pharmaceutical Chemistry, University of Szeged, Eötvös u. 6, H-6720 Szeged, Hungary; szatmari.istvan@pharm.u-szeged.hu (I.S.); lorinczi.balint@pharm.u-szeged.hu (B.L.); fulop@pharm.u-szeged.hu (F.F.); 4 Stereochemistry Research Group of the Hungarian Academy of Sciences, Eötvös utca 6, H-6720 Szeged, Hungary; 5 Institute of Pharmaceutical Chemistry, Interdisciplinary Excellence Centre, University of Szeged, Eötvös u. 6, H-6720 Szeged, Hungary; 6 MTA-SZTE Neuroscience Research Group, Semmelweis u. 6, H-6725 Szeged, Hungary

**Keywords:** kynurenic acid, Mannich reaction, neuroprotection, excitatory amino acid receptors

## Abstract

Kynurenic acid (KYNA), a metabolite of tryptophan, as an excitatory amino acid receptor antagonist is an effective neuroprotective agent in case of excitotoxicity, which is the hallmark of brain ischemia and several neurodegenerative processes. Therefore, kynurenine pathway, KYNA itself, and its derivatives came into the focus of research. During the past fifteen years, our research group has developed several neuroactive KYNA derivatives, some of which proved to be neuroprotective in preclinical studies. In this study, the synthesis of these KYNA derivatives and their evaluation with divergent molecular characteristics are presented together with their most typical effects on the monosynaptic transmission in CA1 region of the hippocampus of the rat. Their effects on the basic neuronal activity (on the field excitatory postsynaptic potentials: fEPSP) were studied in in vitro hippocampal slices in 1 and 200 μM concentrations. KYNA and its derivative **4** in both 1 and 200 μM concentrations proved to be inhibitory, while derivative **8** only in 200 μM decreased the amplitudes of fEPSPs. Derivative **5** facilitated the fEPSPs in 200 μM concentration. This is the first comparative study which evaluates the structural and functional differences of formerly and newly developed KYNA analogs. Considerations on possible relations between molecular structures and their physiological effects are presented.

## 1. Introduction

Glutamate is the main excitatory neurotransmitter in the central nervous system (CNS) that acts both on ionotropic and metabotropic receptors. The excessive glutamate level results in excitotoxicity which is the hallmark of several neurodegenerative processes. Excitotoxicity may result from acute events like stroke or traumatic brain injury [1,2,3]. On the other hand, it plays a role in several neurological diseases [4].

In case of high glutamate level, the receptors of excitatory amino acids (EAARs) such as *N*-methyl-d-aspartate ionotropic glutamate receptor (NMDAR), alfa-amino-3-hydroxy-5-methyl-4-isoxazolepropionic acid (AMPAR), and kainate receptors are overactivated, leading to neuronal damage. The overactivation of these receptors can lead to dysregulation of Ca^2+^ homeostasis, triggering the production of free radicals, oxidative stress, mitochondrial dysfunctions, and finally cell death [5]. The NMDAR is a ligand-gated ion channel that plays an outstanding role in these pathological processes; therefore, it is a potential therapeutic target [6].

Several preclinical studies showed that the attenuation of excitotoxicity may be achieved by blockage of EAARs such as NMDAR [7,8,9,10]. Although it is clear that NMDAR antagonists, such as MK-801, prevents excitotoxicity [11,12], their clinical use is very limited, as a complete NMDAR blockade resulting from their usage can cause serious side effects such as nausea, vomiting, and memory impairment [13]. Additionally, NMDAR blockers may produce psychosis and symptoms of schizophrenia in humans, while in laboratory animals it can induce ataxia and stereotypy [14,15,16]. However, *N*-methyl-d-aspartate receptor (NR2B) subunit-specific antagonists glycine (Gly) and polyamine site agents and ion channel blockers with low affinity got into the focus of research, as they have attenuated the side effects [17]. This was the reason why kynurenic acid (KYNA), an intermediate of the tryptophan metabolism [18,19], also came into the interest of researchers. KYNA is the only known endogenous NMDAR blocker (beside Mg^2+^) that also has broad-spectrum targets: it inhibits NMDARs at Gly-binding site [20] and it can noncompetitively inhibit α7-nicotinic acetylcholine receptors [21], and through this, it can moderate Glu release presynaptically [22]. KYNA has been proposed as the endogenous agonist of G protein-coupled receptor 35 (GPR35), a Gi-protein receptor [23], and a nuclear receptor, called aryl hydrocarbon receptor (AHR) [24]. In addition to the direct effects of KYNA on glutamatergic neurotransmission, an indirect inverse regulation between striatal KYNA and dopamine (DA) levels has also been raised [25].

Reviewing the wide spectrum of effects of KYNA, it can be concluded that KYNA proved to be a neuroprotective substance in most preclinical studies [26,27,28]. This has stimulated the intense study of KYNA itself and of its derivatives, design, and development of new KYNA analogs, prodrugs, and derivatives [18,29,30].

More than fifteen years ago, our research group also started to develop new KYNA derivatives. One of our molecules (**4**), a KYNA amide derivative *N*-(*N*’,*N*’-dimethylaminoethyl)-4-hydroxy-4-quinoline-2-carboxamide hydrochloride), showed electrophysiological properties similar to KYNA in in vitro studies [31], proved to be protective in a transgenic animal model of Huntington’s disease [32] and also in many other preclinical studies [33,34,35,36].

In this article, the synthesis of neuroactive KYNA derivatives and their basic electrophysiological effects studied in in vitro are presented. The tests were carried out in normal artificial cerebrospinal fluid (ACSF).

## 2. Results

### 2.1. Design and Development of KYNA Derivatives

**Scheme 1:**
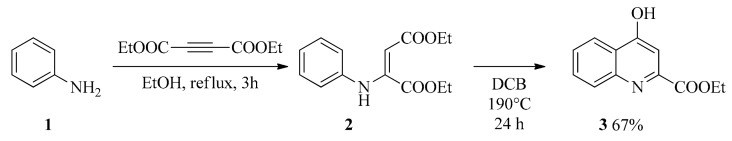
Syntheses of KYNA ethyl ester.

The synthesis of the precursor ethyl 4-hydroxyquinoline-2-carboxylate (**3**) has already been published [37]. During that process, aniline was reacted with diethyl acetylenedicarboxylate, forming the intermediate enamine. In the second ring-closing reaction in diphenyl ether at 250 °C led to the formation of the ethyl ester in an overall yield of 58%. In our case, the following optimizations were applied: (i) After the formation of the enamine **2**, column chromatography was used to purify the intermediate; (ii) Diphenyl-ether was replaced by 1,2-dichlorobenzene that has lower boiling point (190 °C) allowing an easier work-up procedure. These latter conditions led to the formation of **3** in an overall yield of 67% (Scheme 1).

**Scheme 2:**
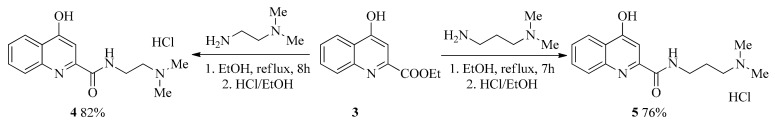
Syntheses of KYNA amide derivatives.

Compounds **4, 5** were synthesized by direct amidation of the ethyl ester of KYNA (**3**) with *N*^1^,*N*^1^-dimethylethane-1,2-diamine (to achieve **4**) or with *N*^1^,*N*^1^-dimethylpropane-1,3-diamine (to achieve **5**) (Scheme 2).

**Scheme 3:**
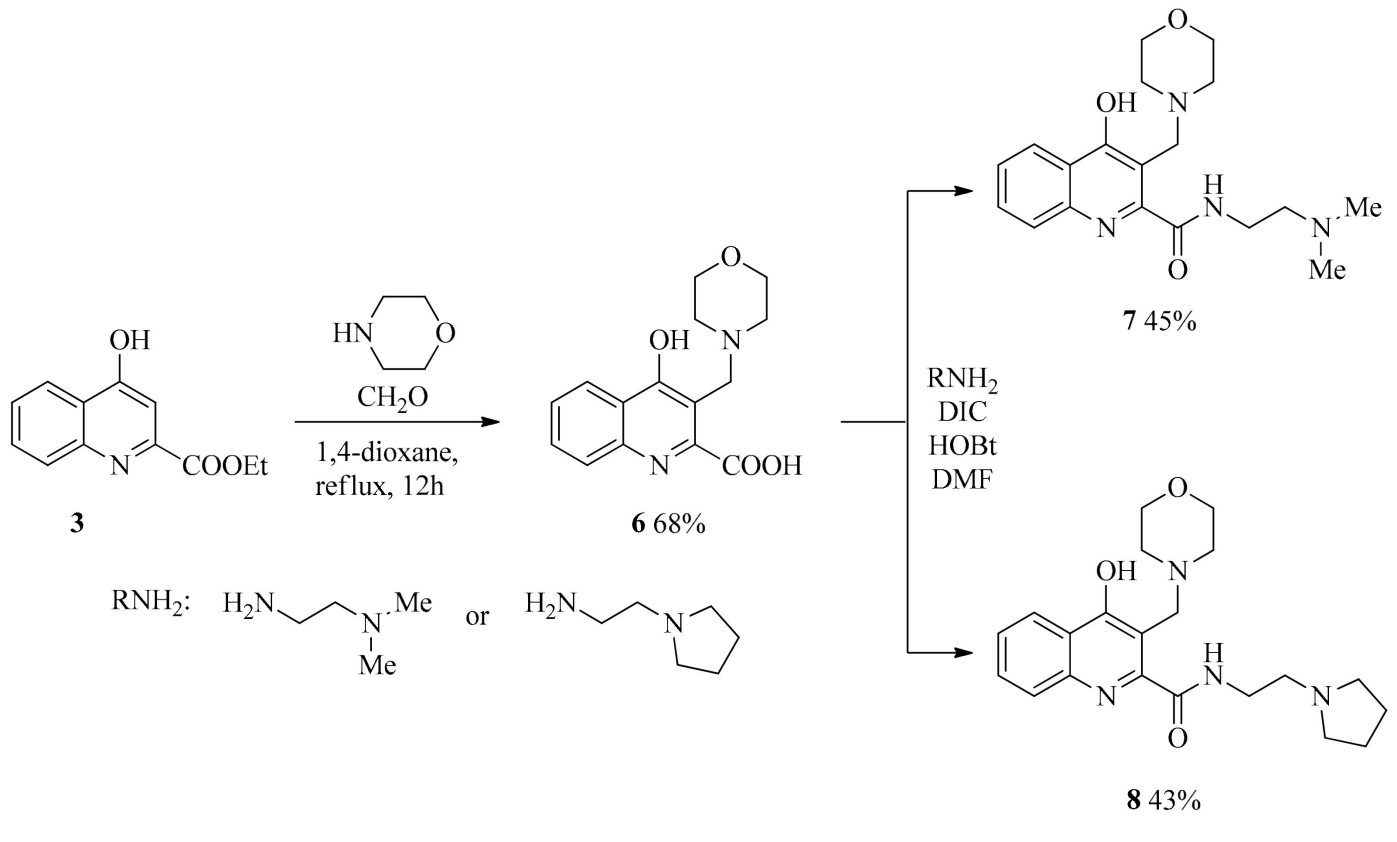
Syntheses of aminoalkylated KYNA amide derivatives.

As previous studies indicated, the addition of a proton acceptable nitrogen is preferable for better biological activity [38]. This can be achieved either through amidation using amines containing tertiary nitrogen (Scheme 2), or via modified Mannich reaction (*m*MR) by using secondary amine derivatives. It has been suspected that the presence of multiple cationic centers together will contribute to the biological effect with greater magnitude. Thus, the syntheses of **7** and **8** containing two such functional groups were planned in a step by step synthetic pathway (Scheme 3). First, the synthesis of **6** was carried out. During the aminoalkylation of **3**, morpholine as a representative secondary cyclic amine with formaldehyde was used to form the iminium salt that would attack the C-3 position on the KYNA skeleton. Afterward, the amidations of **6** were achieved in dimethylformamide through the activation of the free carboxylic group with *N*,*N*′-diisopropylcarbodiimide (DIC). During the reactions, 1-hydroxybenzotriazole hydrate (HOBt) was also used to achieve an active-ester intermediate and further improve the yield (Scheme 3).

### 2.2. Electrophysiology

**Figure 1:**
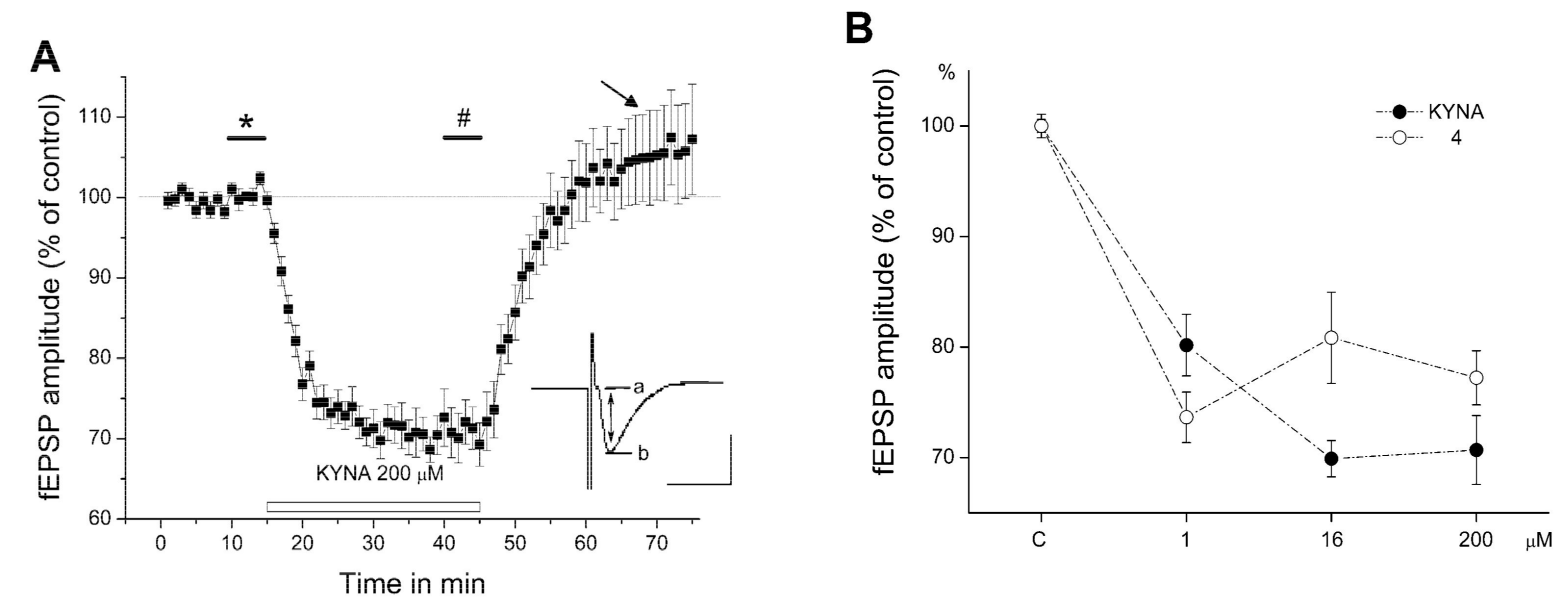
Effect of KYNA and **4** (one of its derivatives) on the field excitatory evoked potentials (fEPSPs) recorded in CA1 region of hippocampus. **A**: KYNA administration (in 200 µM concentration) for 30 min resulted in decrease of the fEPSPs’ amplitudes reversibly. During wash-out, the amplitudes not only recovered but also were facilitated in the recorded period (arrow) *: samples for the baseline were taken during the last 5 min of control period. #: samples for calculation of the effect were taken during the last 5 min of wash-in period. Inset: an original fEPSP. Amplitudes were measured from ‘a’ to ‘b’. Calibration: 20 ms and 0.5 mV. **B**: dose-dependent responses to KYNA and **4** in concentrations 1 µM, 16 µM, and 200 µM, respectively. In case of KYNA, *n* = 7 at 1 μM and 16 μM and *n* = 10 at 200 μM. In case of **4** *n* = 10 at 1 μM and 16 μM and *n* = 8 at 200 μM.

Schaffer collateral-stimulation results in field excitatory postsynaptic potentials (fEPSPs) in the pyramidal layer of region CA1 of the hippocampus with 0.5–0.8 mV amplitudes (Figure 1A, inset). Administration of KYNA or the presented KYNA derivatives in 200 μM concentration resulted in either decrease or increase in the amplitudes of the fEPSPs. KYNA derivatives (except **4** and **5**) applied in 1 μM concentration induced less unequivocal and significant changes.

**Figure 2:**
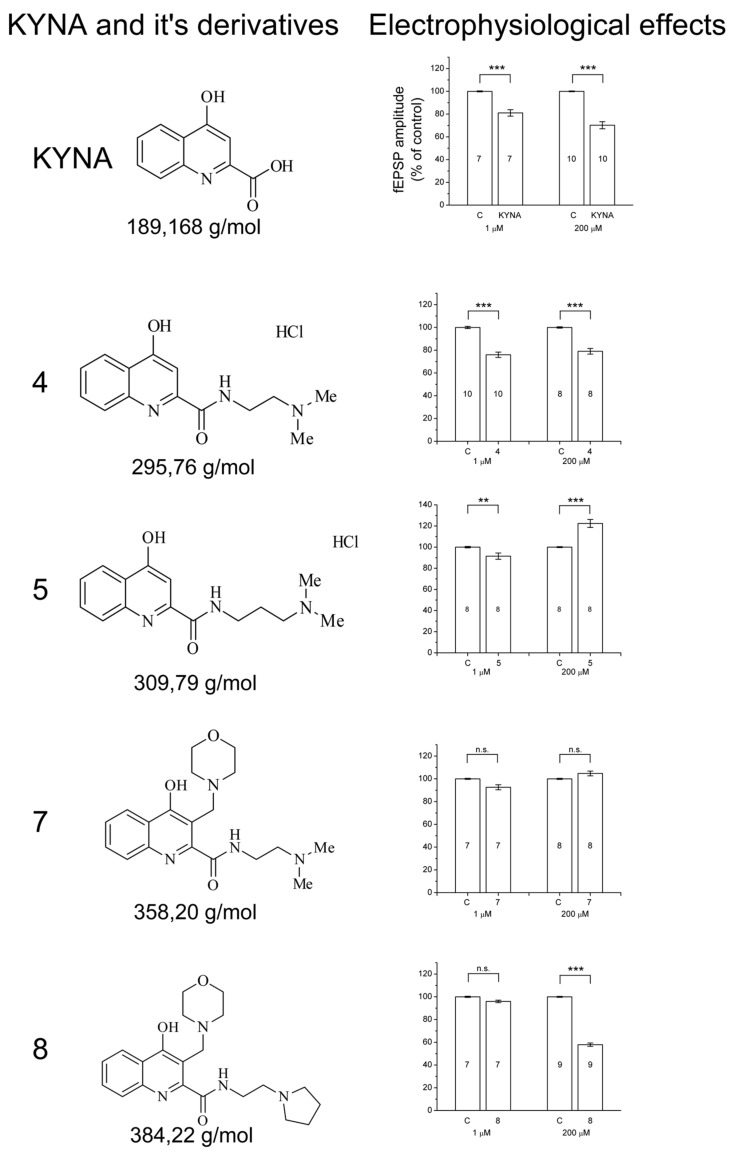
Structure and molecular weight of KYNA and of its derivatives (**4**, **5**, **7**, **8**), respectively, together with their effects on the field excitatory postsynaptic potentials (fEPSPs) recorded in CA1 region of hippocampus. Control (c) was observed during the last 5 min of resting period, before administration of a compound (* in Figure 1A). Effect on the amplitudes of fEPSPs was observed during the last 5 min of wash-in periods, respectively (# in Figure 1A). Each slice was subjected to only one measurement. Changes in fEPSP amplitudes (mean ± SEM) due to application of KYNA or of its analogs are shown in the bar graphs. The numbers of measurements (N) are indicated in the columns; n.s. = *p* > 0.05; **p < 0.01; ***p < 0.001).

Following the control period, an immediate decrease in fEPSP amplitudes could be detected at the wash-in period of KYNA (200 μM) (Figure 1A). The maximal reduction was 25%–30% compared to controls, which lasted until the end of wash-in (Figure 1A). During wash-out period, amplitudes started to rise immediately, and after 20–25 min, they became stable, showing a 6%–8% increase compared to the controls (arrow in Figure 1A). KYNA and **4** (a KYNA amide), both in 1 µM and 200 µM concentrations, resulted in significant decrease in the amplitude (Figure 2 KYNA and compound **4**). To show the effect of **4** in comparison with KYNA in more detailed, dose–effect relations are presented (Figure 1B). The effect of **5** is fundamentally different. Its administration at 200 μM concentration resulted in 15%–25% facilitation of fEPSPs amplitudes, while in 1 μM concentration its effect was slightly inhibitory (Figure 2, compound **5**).

Though the structures of **7** and **8** are similar to each other, their effects are different. Administration of **7** in 200 μM concentration induced a slight increase in the amplitudes of fEPSPs during the wash-in period, while in 1 μM, it had a weak inhibitory effect, but none of them proved to be significant (Figure 2, compound **7**). In case of **8**, the results acquired during the administration were not stable. In most cases it (in 200 μM) resulted in a serious decrease in amplitudes (to 60% of controls, e.g., Figure 2, compound **8**), and in few cases, the inhibitory effect of **8** was smaller. In 1 μM concentration, it had hardly any effect.

## 3. Discussion

A mass of early results from other laboratories [39,40,41] and our own previous studies demonstrated that neuroprotection can be achieved not only with KYNA but also by administration of either kynurenine (KYN, the prodrug of KYNA) or KYNA derivatives [10,26,42,43,44,45]. During the past one and half decade, we have made great efforts to develop new KYNA derivatives which are neuroactive (and hopefully protective in such pathological states as ischemia or in the models of different neurological diseases) [28,30,46].

In this study, we focused on four neuroactive KYNA derivatives; keeping in mind the structure of molecules and the effects of substances on the neuronal transmission in a relatively simple monosynaptic preparation (Schaffer collateral/commissural pathway - pyramidal cells). The induced fEPSPs well demonstrate the neural signaling, in which mainly NMDA and AMPA receptors are involved [47,48].

The electrophysiological studies were carried out both in lower and higher concentrations (1 and 200 μM, respectively). While KYNA and **4** decreased the amplitudes effectively in both concentrations, the effect of derivatives **5**, **7**, and **8** in 1 μM concentration was insignificant. In this study, we focused on the 200 μM concentration because in another series of experiments, aiming to study the phenomenon of neuroprotection, that concentration was used. KYNA administration resulted in reduction in amplitudes of fEPSPs, which can be well explained by its binding to the strychnine-insensitive glycine binding site of the NMDA receptor [20]. After KYNA application, during the wash-out period, the fEPSPs not only recovered but they were also augmented. Looking for explanation, one can think of the results of Prescott et al., who found that KYNA has a dual action on AMPA receptors [49]. Similarly, we found that KYNA has a Janus-faced effect on fEPSPs; in micromolar range it has inhibitory, while in nanomolar range it has facilitatory effect [50]. This might be based on that during the wash-out period the KYNA concentration gradually decreased to nanomolar range.

The phenomenon was true for **4** as well; significant decrease in amplitudes of fEPSPs was observed during its administration in all studied concentrations. To demonstrate the similar inhibitory properties of KYNA and **4**, their dose-dependent responses were presented (Figure 1B). Similar result was published by Marosi et al. [31]. **4** was our firstly synthesized KYNA amide, effective as an inhibitor of fEPSPs in in vitro studies, and it proved to be outstandingly effective in earlier studies of neuroprotection [31,32,43,51]. It was effective also in systemic administration, which suggests that **4** might penetrate the blood-brain barrier (BBB) much more effectively than KYNA [30]. It can be explained by the molecular structure of **4** as it has a side chain containing cationic center that may help the penetration through the BBB and through the creation of hydrochloride salt it can grant water solubility.

Contrary to the small structural difference (**5** differs from **4** only in a -CH_2_- in the side chain), the effect of **5** is fundamentally different; its administration (in 200 μM) did not induce inhibition of fEPSPs but facilitated the amplitudes (Figure 2, compound **5**). Similar results were also found in the early tests of these molecules made on anesthetized animals [30].

The difference between the molecular structures of **7**, **8** and **4**, **5** is that the latter compounds are unsubstituted at position 3, while **7** and **8** are aminoalkylated at C-3 of the KYNA skeleton. Though **7** and **8** have similar structure (the only difference between the two molecules is the cationic center of the amide side chain), their effects (in 200 μM) were fundamentally different; **7** resulted in a slight but insignificant facilitation while **8** in most cases induced a marked inhibition of fEPSP amplitudes (Figure 2, compounds **7** and **8**).

Although it is hard to discuss comprehensive correlations between structure and biological effects, we could determine certain structural changes leading to different biological activity. Compound **4**, as previously investigated, shows similar effect as KYNA; however, the insertion of an additional -CH_2_-group in the amide sidechain (compound **5**) resulted in an antagonist effect. Similar results were observed during the evaluation of compound **7** that bears the amide sidechain of **4** and an additional aminoalkyl group at C-3. These results mean that the insertion of an additional cationic center at position 3 has a characteristic influence on the electrophysiological effects.

The explanation of the phenomenon is not clear. Probably, it cannot be elucidated simply with the binding to the synaptic AMPA- and/or NMDA receptors. It is known that beside the synaptic glutamatergic receptors, perisynaptic and extrasynaptic receptors exist, which are distinct from each other [52,53,54,55]. Basically, according to the subunit composition, in adults, GluN2A subunits of NMDA receptors are predominantly synaptic, GluN2B-containing receptors are mostly extrasynaptic, though it is not true in all cases, for example, GluN2B subunits are also located in synapses in several brain regions [56]. Activation of these extrasynaptic receptors leads to changes in excitability of neurons and take part in several pathological processes like excitotoxicity, neurodegeneration, or neuroinflammation [57]. It might be that the amplitudes’ changes involve different mechanisms.

Our most effective molecules are KYNA amides, which are known to be capable of the selective inhibition of the NR2B subunit-containing NMDA receptors [58]. However, the effects of different neuroactive KYNA derivatives have never been compared keeping in mind their structure. This is the first comparative study which evaluates the structural and functional differences of formerly and newly developed KYNA analogs. Unfortunately, at this moment, we do not know more details about the binding of these molecules to the different synaptic and extrasynaptic glutamatergic receptors, but the results of these preclinical studies are promising, as these KYNA derivatives might be promising candidates for clinical trials.

## 4. Materials and Methods

### 4.1. Drugs

KYNA was from Sigma-Aldrich (Steinheim, Germany). Compounds (**5**, **7**, **8**) in these studies were used at final concentration of 1 and 200 μM, respectively, while KYNA and **4** in concentrations of 1, 16, and 200 μM, dissolved in normal ACSF. The pH of the solutions was continuously monitored and kept at 7.4.

### 4.2. Synthesis

The ^1^H and ^13^C spectra were recorded in DMSO or D_2_O solutions in 5 mm tubes, at room temperature, with a Bruker spectrometer (Karlsruhe, Germany) at 500 (^1^H) and 125 (^13^C) MHz, with the deuterium signal of the solvent as the lock and TMS as internal standard.

Melting points were determined on a Hinotek X-4 melting point apparatus (Ningbo, China). Elemental analyses were performed with a Perkin-Elmer 2400 CHNS elemental analyzer (Waltham, MA, USA). Merck Kieselgel 60F254 plates (Budapest, Hungary) were used for TLC. The microwave reactions were performed with a CEM Discover SP microwave reactor (Matthwes, NC, USA).

#### 4.2.1. General Procedure for the Synthesis of Amides (4, 5)

Ethyl 4-hydroxyquinoline-2-carboxylate (**3**) (1.0 mmol), *N*^1^*,N*^1^-dimethylethane-1,2-diamine (2.0 mmol, to achieve **4**), or *N*^1^*,N*^1^-dimethylpropane-1,3-diamine (2.0 mmol, to achieve **5**) were placed in a 50 mL round bottom flask. The mixture was refluxed in 30 mL EtOH for 7–8 h. After the evaporation of the solvent, the residues were crystallized with Et_2_O (10 mL). The products were dissolved in 15 mL of EtOH and HCl/EtOH (22%) was added until reaching pH = 1. Et_2_O (10 mL) was added to the solution until precipitation. The crystals were filtered and washed with 2 × 15 mL Et_2_O yielding **4** or **5,** respectively.

*N-(2-(dimethylamino)ethyl)-4-hydroxyquinoline-2-carboxamide hydrochloride* (**4**). Yield: 241 mg (82%); m.p. 150–153 °C. ^1^H-NMR (D_2_O); 3.03 (6H, s); 3.50 (2H, t, J = 6.0 *Hz*); 3.88 (2H, t, J = 6.1 *Hz*); 6.80 (1H, s); 7.54 (1H, t, J = 7.4 *Hz*); 7.76 (1H, d, J = 8.3 *Hz*); 7.83 (1H, t, J = 7.9 *Hz*); 8.12 (1H, d, J = 8.3 *Hz*); ^13^C-NMR (D_2_O); 35.3; 43.2; 53.4; 106.8; 119.4; 124.3; 124.7; 125.8; 134.0; 139.4; 141.7; 163.7; 180.3; ppm. C_14_H_18_ClN_3_O_2_ (295.76): calcd. C, 56.80; H, 6.09; N, 14.20; found C, 56.73; H, 6.08; N, 14.16.

*N-(3-(dimethylamino)propyl)-4-hydroxyquinoline-2-carboxamide* (**5**). Yield: 238 mg (77%); m.p. 222–226 °C. ^1^H-NMR (D_2_O); 2.09–2.17 (2H, m); 2.95 (6H, s); 3.28 (2H, t, J = 8.6 *Hz*); 3.57 (2H, t, J = 6.7 *Hz*); 6.86 (1H, s); 7.58 (1H, t, J = 7.2 *Hz*); 7.80 (1H, d, J = 8.0 *Hz*); 7.86 (1H, t, J = 7.1 *Hz*); 8.16 (1H, d, J = 8,3 *Hz*); ^13^C-NMR (D_2_O); 24.0; 37.1; 42.9; 55.4; 106.1; 119.5; 124.1; 124.2; 126.3; 134.3; 139.3; 142.5; 162.9; 179.0 ppm. C_15_H_20_ClN_3_O_2_ (309.79): calcd. C, 58.10; H, 6.46; N, 13.56; found C, 58.14; H, 6.42; N, 13.53.

*4-hydroxy-3-(morpholinomethyl)quinoline-2-carboxylic acid* (**6**). Morpholine (174 mg, 2.0 mmol), ethyl 4-hydroxyquinoline-2-carboxylate (**3**; 217 mg, 1.0 mmol), 22% aqueous formaldehyde (410 mg, 3.0 mmol), and 30 mL of 1,4-dioxane were placed in a 50 mL round bottom flask. The mixture was refluxed for 6 h. After the evaporation of the solvent, the residue was dissolved in 40 mL water and extracted with 3 × 30 mL of DCM. The collected organic phases were dried (Na_2_SO_4_), the solvent was removed by evaporation, and the residue was crystallized from 15 mL *n*-hexane:EtOAc (4:1) to yield **6**. Yield: 196 mg (68%); m.p. 253–256 °C. ^1^H-NMR (DMSO); 3.16–3.40 (4H, m); 3.66–3.84 (2H, m); 3.84–4.01 (2H, m); 4.56 (2H, s); 7.43 (1H, t, J = 7.4 *Hz*); 7.75 (1H, t, J = 7,9 *Hz*); 8.04 (1H, d, J = 8.0 *Hz*); 8.14 (1H, d, J = 7.9 *Hz*); 10.39 (1H, brs); 12.18 (1H, brs); ^13^C-NMR (DMSO); 52.0; 52.2; 64.1; 110.7; 120.5; 125.2; 125.5; 125.9; 133.8; 139.9; 143.3; 164.6; 178.2 ppm. C_15_H_16_N_2_O_4_ (288.30): calcd. C, 62.43; H, 5.55; N, 9.71; found C, 62.38; H, 5.56; N, 9.75.

#### 4.2.2. General Procedure for the Syntheses of Aminoalkylated Amides 7, 8

The 4-hydroxy-3-(morpholinomethyl)quinoline-2-carboxylic acid **6** (1.0 mmol) with *N*^1^*,N*^1^-dimethylethane-1,2-diamine (2.0 mmol to achieve **7**) or 2-(pyrrolidin-1-yl)ethanamine (2.0 mmol to achieve **8**) were dissolved in DMF (20 mL). The reaction was cooled to −10 °C while HOBt (2.0 mmol) was added and the solution was stirred for 10 min. *N*,*N*′-diisopropylcarbodiimide (2.0 mmol) was added. After reaching room temperature, the mixture was stirred for additional 7–10 h. After the evaporation of the solvent, the residue was dissolved in 20 mL DCM and was washed with 2 × 20 mL H_2_O. The organic phase was dried with Na_2_SO_4_ and the solvent was removed under reduced pressure. The residue was crystallized using Et_2_O (10 mL) yielding **7** or **8,** respectively.

*N-(2-(dimethylamino)ethyl)-4-hydroxy-3-(morpholinomethyl)quinoline-2-carboxamide* (**7**). Yield: 161 mg (45%); m.p. > 350 °C. 2.19 (6H, s); 2.41–2.48 (6H, m); 3.45–3.52 (2H, m); 3.53–3.59 (4H, m); 3.60 (2H, s) 7.31 (1H, t, J = 7.3 Hz); 7.64 (1H, t, J = 7.7 Hz); 7.86 (1H, d, J = 8.3 Hz); 8.08 (1H, d, J = 8.1 Hz); 10.63 (1H, brs); ^13^C-NMR (DMSO); 38.4; 45.8; 51.0; 52.4; 59.1; 67.0; 113.7; 120.4; 124.4; 124.8; 126.0; 131.0; 132.8; 140.5; 163.9; 177.8; ppm; C_19_H_26_N_4_O_3_ (358.43): calcd. C, 63.61; H, 7.25; N, 15.62; found C, 63,66; H, 7.26; N, 15.65.

*4-hydroxy-3-(morpholinomethyl)-N-(2-(pyrrolidin-1-yl)ethyl)quinoline-2-carboxamide* (**8**). Yield: 165 mg (43%); m.p. > 350 °C. ^1^H-NMR (DMSO); 1.63–1.73 (4H, m); 2.46–2.52 (6H, m); 2.64 (2H, t, J = 6.2 Hz); 3.35–3.41 (2H, m); 3.48–3.63 (8H, m); 7.34 (1H, t, J = 7.1 Hz); 7.66 (1H, t, J = 7.2 Hz); 7.88 (1H, d, J = 8.0 Hz); 8.09 (1H, d, J = 8.0 Hz); 10.82 (1H, brs); 11.78 (1H, brs); ^13^C-NMR (DMSO); 23.6; 50.6; 52.2; 54.1; 55.4; 66.6; 113.4; 119.6; 124.1; 124.3; 125.7; 132.6; 139.7; 144.2; 163.2; 177.6 ppm; C_21_H_28_N_4_O_3_ (384.47): calcd. C, 65.54; H, 7.28; N, 14.56; found C, 65.50; H, 7.27; N, 14.55.

### 4.3. Animals and Housing Conditions

Recordings were made from brain slices taken from 20- to 28-day-old male Wistar rats (N = 62) supplied by Charles River Laboratories (Wilmington, MA, USA). Animals were kept under constant environmental conditions (23 Celsius degree; humidity 55 ± 5%; a 12-h/12-h light/dark cycle) and were housed individually in standard plastic cages, where they had free access to food and water. Every effort was made to minimize the number of animals used and their suffering. The principles of animal care (NIH publication No. 85–23) and the protocol for animal care approved both by the Hungarian Health Committee (1998) and by the European Communities Council Directive (2010/63/EU) were followed. Ethical license: XX./842/2016. Before the experimental procedures, all the rats were in normal health and had no neurological deficits.

### 4.4. Slice Preparation

Coronal slices (350 µm), prepared from the middle part of their hippocampi with a vibratome (Leica VT1200S, Wetzlar, Germany), were kept in an ice-cold artificial cerebrospinal solution (ACSF) composed of (in mM) 130 NaCl, 3.5 KCl, 1 NaH_2_PO_4_, 24 NaHCO_3_, 1 CaCl_2_, 3 MgSO_4_, and 10 D-glucose (all from Sigma, Germany), saturated with 95% O_2_ + 5% CO_2_. 3 slices were made from the brain of an animal. The slices were then transferred to a slice holder chamber, where they were warmed up to 32 °C shortly, and then kept 19–20 °C until usage to allow the slices to recover in the solution used for recording (differing only in that it contained 3 mM CaCl_2_ and 1.5 mM MgSO_4_). The flow rate in the Haas-type recording chamber was 1.5–2 mL/min and the experiments were performed at 34 °C.

### 4.5. Electrophysiology

In order to obtain orthodromic stimulation of the Schaffer collateral/commissural pathway, the stimulating electrode (a bipolar concentric stainless steel electrode developed by Neuronelektrod Ltd., Hungary) was placed in the stratum radiatum between the CA1 and CA2 regions. The stimulus intensity was adjusted between 20 and 50 µA (constant current, 0.2-ms pulses delivered at 0.05 Hz) to evoke the half-maximal response. Field excitatory postsynaptic potentials (fEPSPs) were recorded with glass micropipettes with a resistance of 1.5–2.5 MΩ filled with ACSF. The recordings were amplified with a neutralized, high-input-impedance preamplifier and filtered (1 Hz–3 kHz). The fEPSPs were digitized (AIF-03, Experimetria Ltd., Budapest, Hungary), acquired at a sampling rate of 10 kHz, saved to a PC, and analyzed off-line with Origin 8.0 software (OriginLab Corporation, Northampton, MA, USA). The fEPSPs were monitored for 15–20 min until the amplitudes were stable. After having stabilized the amplitudes, the last 5 min of resting period (before administration of a compound) were taken as baseline (control, see * in Figure 1A). After baseline recording, KYNA or its analogs were bath-applied for 30 min, and a wash-out occurred for another 30-min-period. The effect of a compound was calculated based on the amplitudes obtained during the last 5 min of wash-in period (see # in Figure 1). The properties of the basal glutamatergic synaptic transmission were also tested by means of fEPSP recordings; in IO (input-output) graphs the fEPSP amplitudes are expressed against gradually increasing stimulating currents (0–60 µA, in 5-µA steps) in certain groups (data not shown). Each slice was subjected to only one measurement [59,60]. 7–10 measurements (N) were carried out in case of each concentration of each compound, respectively. The actual numbers of measurements (N) are in the bars of graphs in Figure 2. Altogether, 117 slices were used to test the effects of KYNA and of its analogs.

## 5. Statistical Analysis

Field excitatory postsynaptic potentials (fEPSPs) evoked by Schaffer collateral stimulation were measured from ‘a’ to ‘b’ (see Figure 1A, inset). Origin 8.0 software (OriginLab Corporation, Northampton, MA, USA) was used for the analyses. Results are expressed as mean ± SEM. One-way repeated-measures ANOVA with Dunnett post hoc tests was used for assessing the differences between amplitudes of controls and of KYNA and of its analogs during wash-in.

## Supplementary Materials

Supplementary File 1
