# Supplementary material for: Structural Evaluation and Electrophysiological Effects of Some Kynurenic Acid Analogs
Source: Molecules. 2019 Sep 26;24(19):3502. doi: 10.3390/molecules24193502 (PMC6803921; doi:10.3390/molecules24193502)

molecules

Supplementary information

Structural Evaluation and Electrophysiological Effects of Some Kynurenic Acid Analogs

Evelin Fehér [1,2,†], István Szatmári [3,4,5,†], Tamás Dudás [2], Anna Zalatnai [2], Tamás Farkas [2,*], Bálint Lőrinczi [3,4], Ferenc Fülöp [3,4,5], László Vécsei [1,6] and József Toldi [2]

[1] Department of Neurology, Interdisciplinary Excellence Centre, Albert Szent-Györgyi Clinical Center, Faculty of Medicine, University of Szeged, Semmelweis u. 6, H-6725 Szeged, Hungary; feher.evelin23@gmail.com (E.F.); vecsei.laszlo@med.u-szeged.hu (L.V.)

[2] Department of Physiology, Anatomy and Neuroscience, University of Szeged, Közép fasor 52, H-6726 Szeged, Hungary; dudi.14t@gmail.com (T.D.); zalatnaianna@gmail.com (A.Z.); toldi@bio.u-szeged.hu (J.T.)

[3] Institute of Pharmaceutical Chemistry, University of Szeged, Eötvös u. 6, H-6720 Szeged, Hungary; szatmari.istvan@pharm.u-szeged.hu (I.S.); lorinczi.balint@pharm.u-szeged.hu (B.L.); fulop@pharm.u-szeged.hu (F.F.)

[4] Stereochemistry Research Group of the Hungarian Academy of Sciences, Eötvös utca 6, H-6720 Szeged, Hungary

[5] Institute of Pharmaceutical Chemistry, Interdisciplinary Excellence Centre, University of Szeged, Eötvös u. 6, H-6720 Szeged, Hungary

[6] MTA-SZTE Neuroscience Research Group, Semmelweis u. 6, H-6725 Szeged, Hungary

[†] These authors contributed equally to this work.

* Correspondence: tfarkas@bio.u-szeged.hu; Tel.: +36 (62) 544-381

Contents:

1. Copies of ^{1}H- and ^{13}CNMR spectra of **2-6** ………………………………………… S2

***N*-(2-(dimethylamino)ethyl)-4-hydroxyquinoline-2-carboxamide hydrochloride (2)**

D_2O

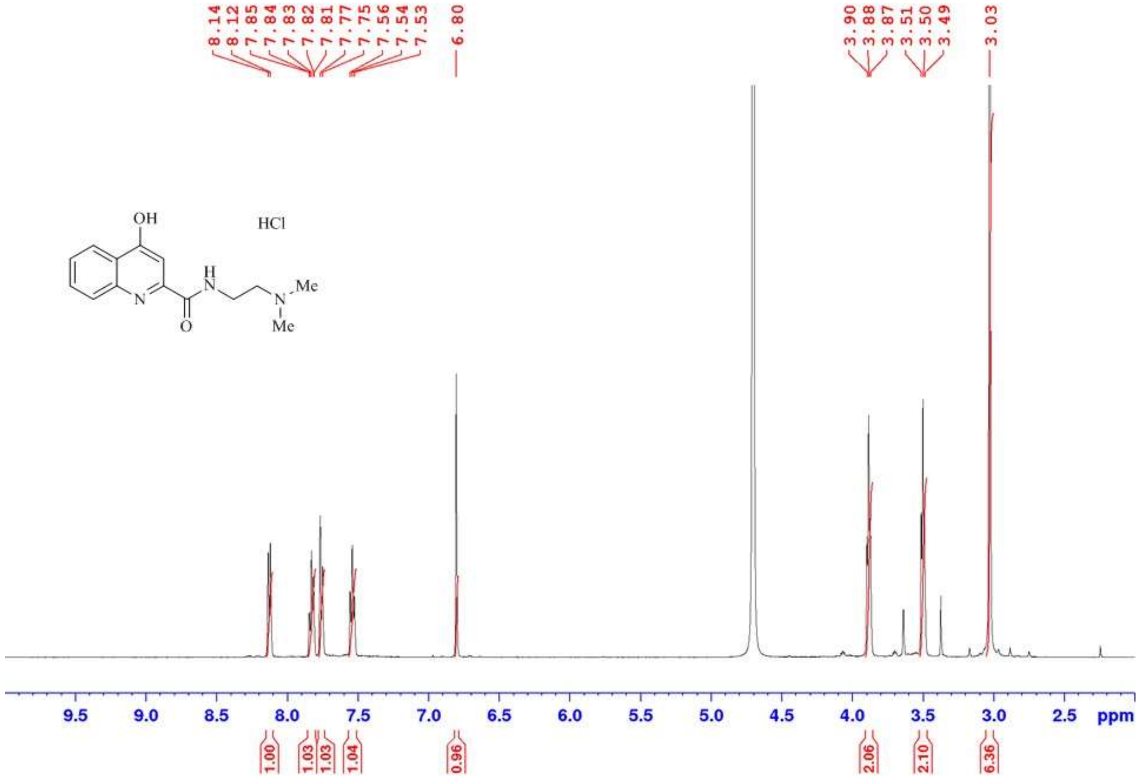

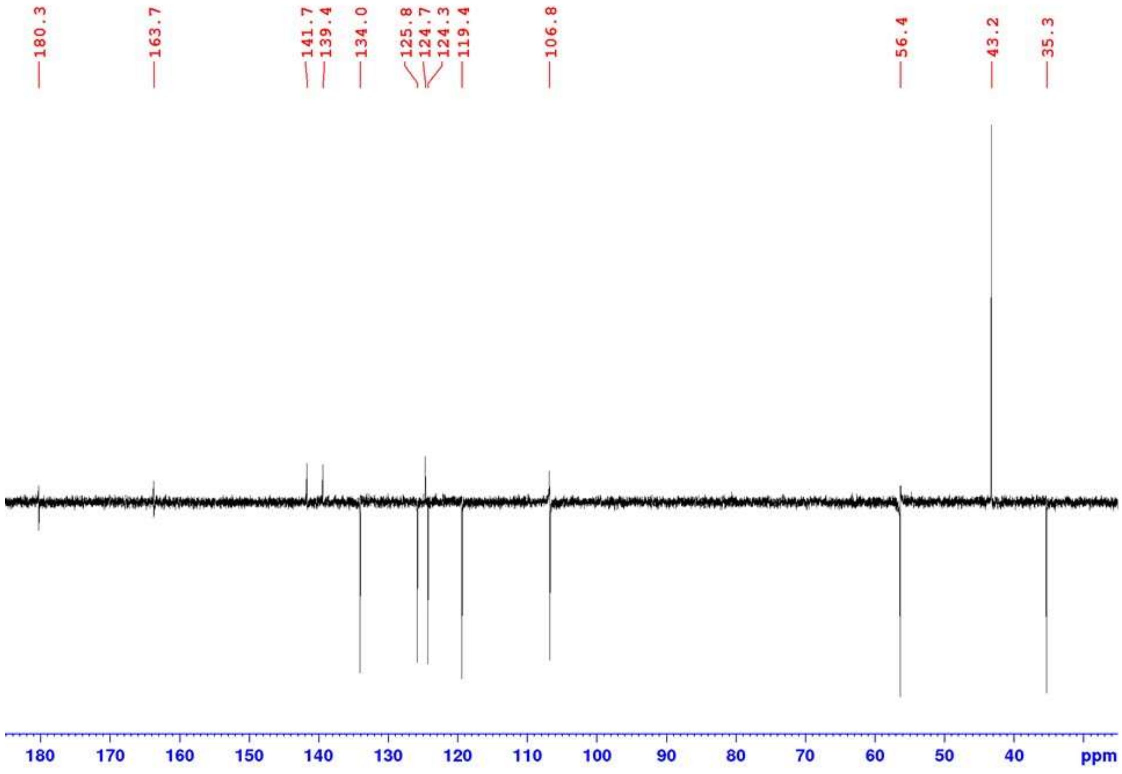

N-(3-(dimethylamino)propyl)-4-hydroxyquinoline-2-carboxamide (3)

D_2O

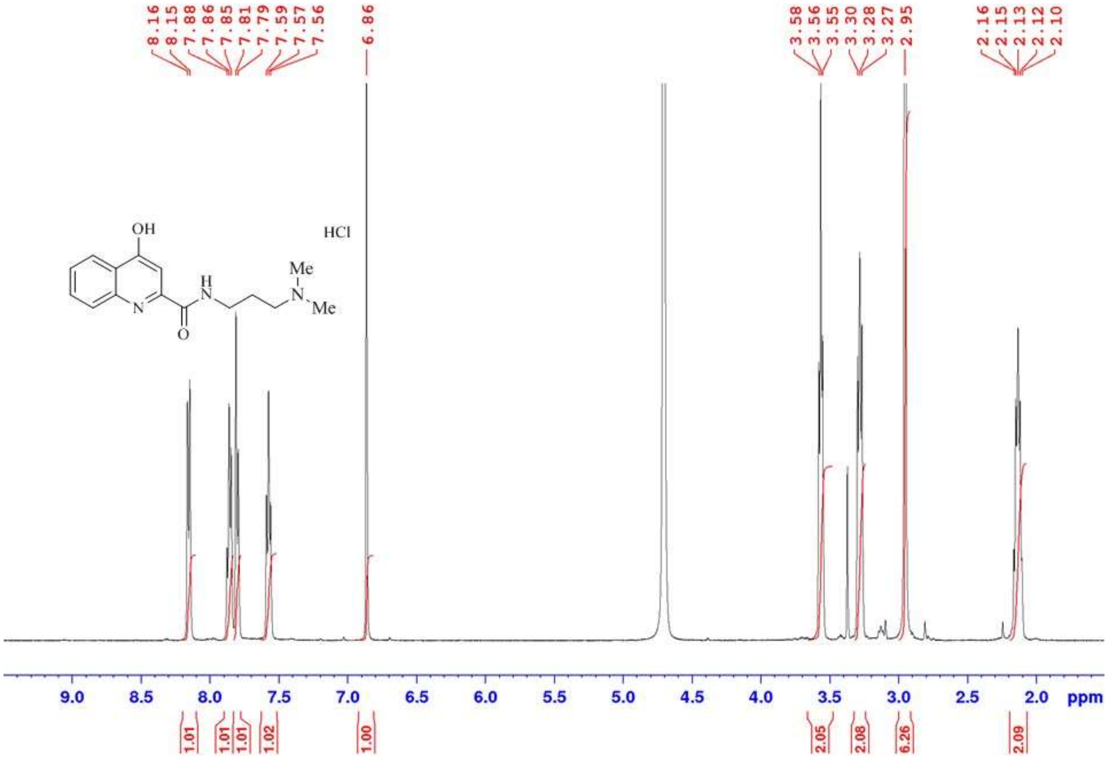

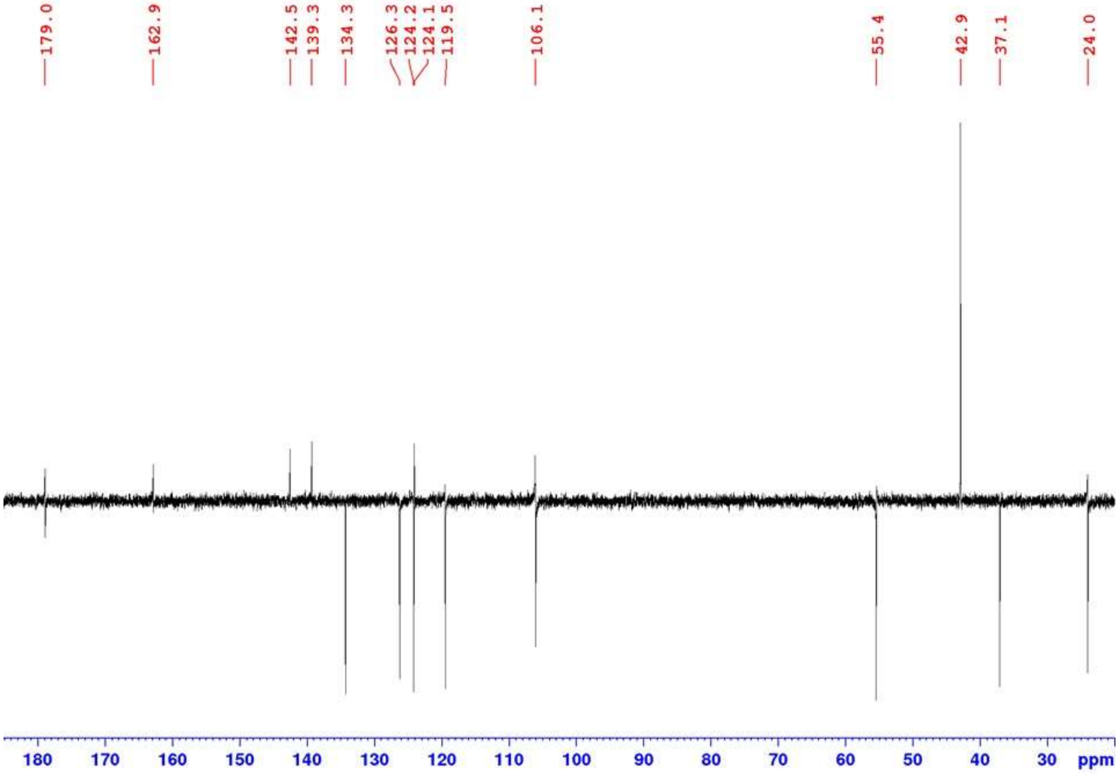

4-hydroxy-3-(morpholinomethyl)quinoline-2-carboxylic acid (4)
DMSO

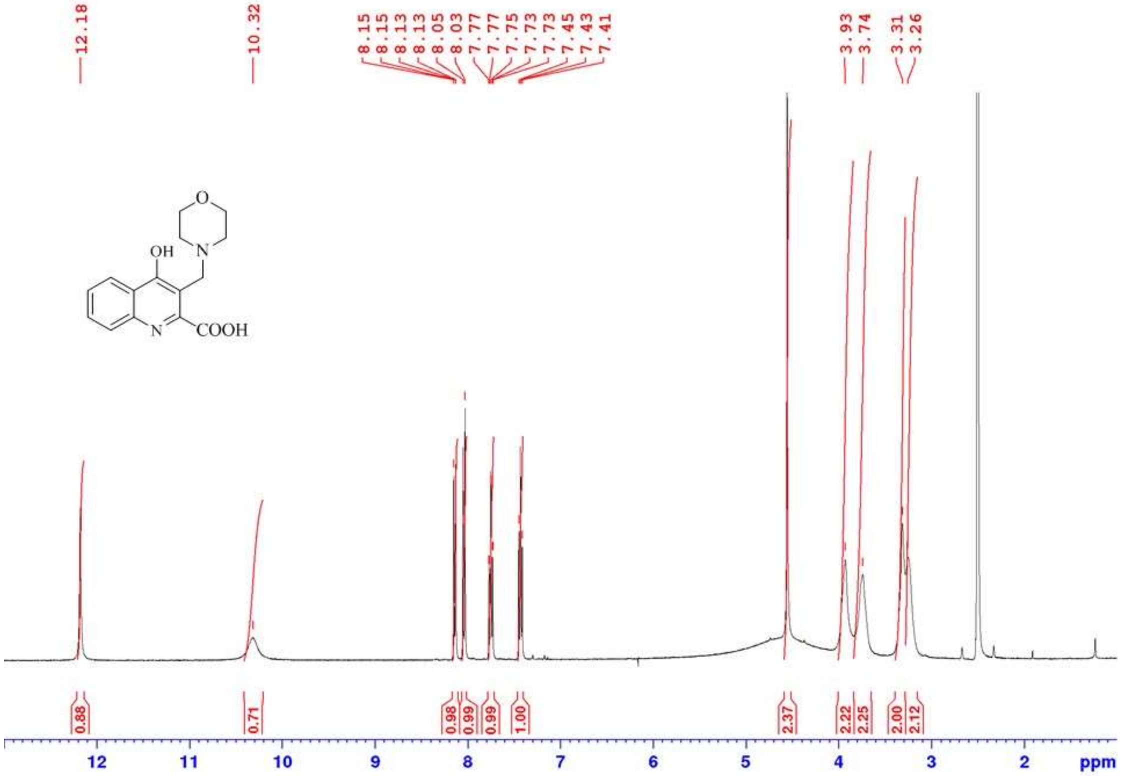

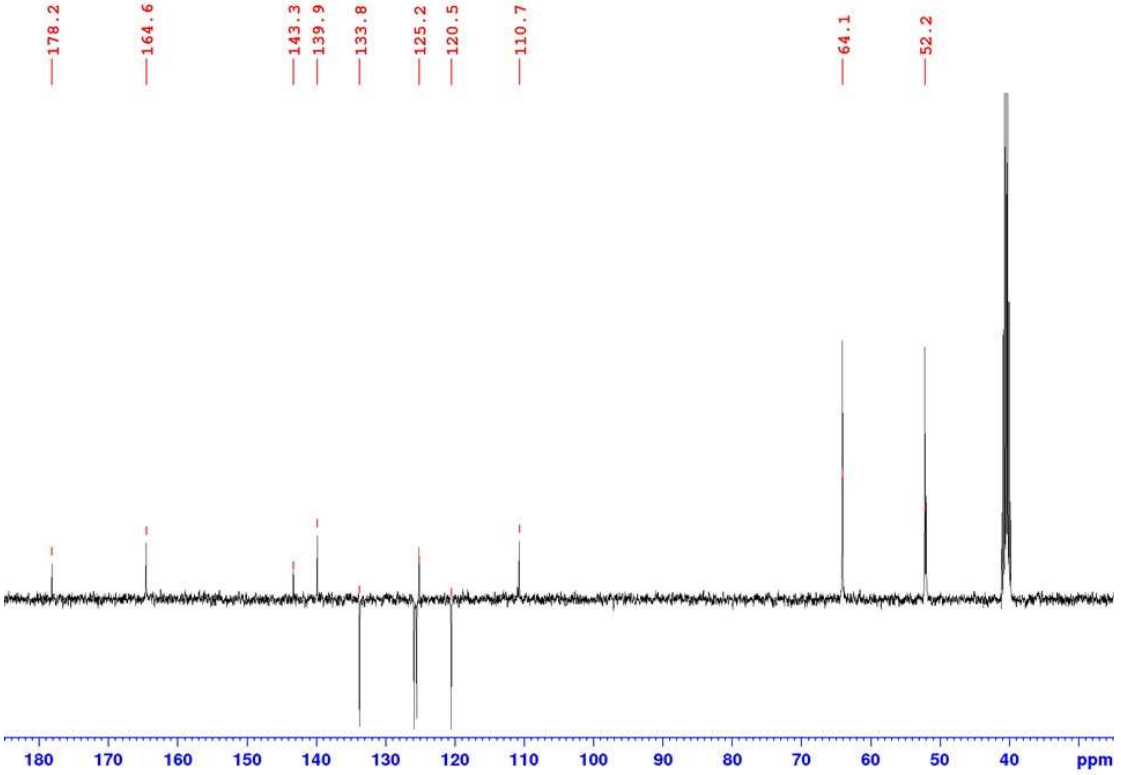

N-(2-(dimethylamino)ethyl)-4-hydroxy-3-(morpholinomethyl)quinoline-2-carboxamide (5)

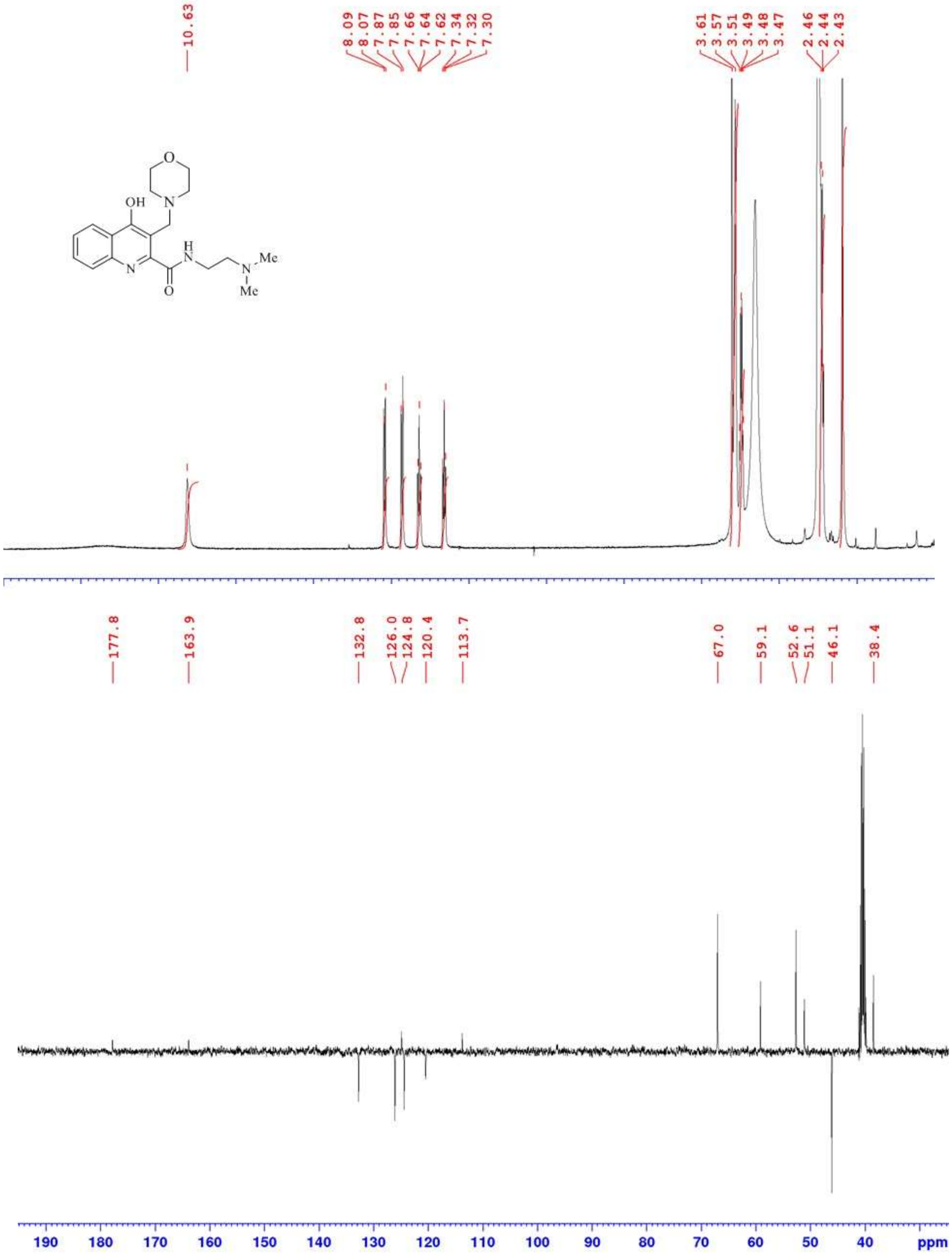

DMSO

4-hydroxy-3-(morpholinomethyl)-N-(2-(pyrrolidin-1-yl)ethyl)quinoline-2-carboxamide (6)
DMSO

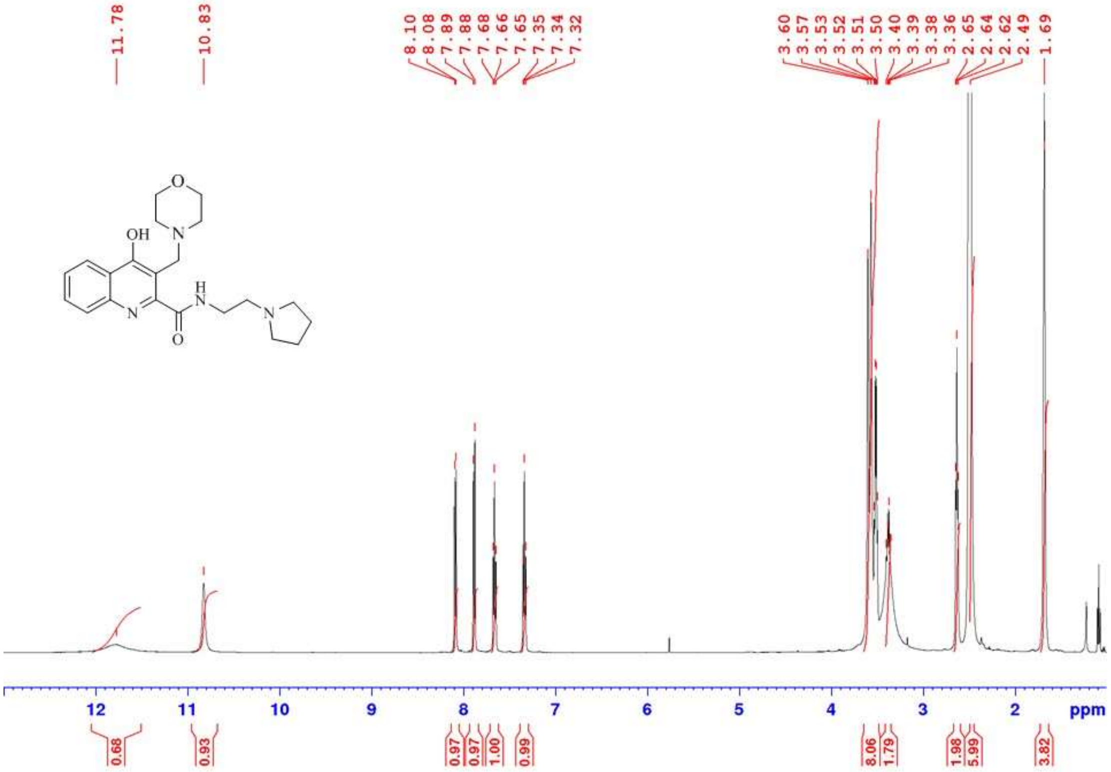

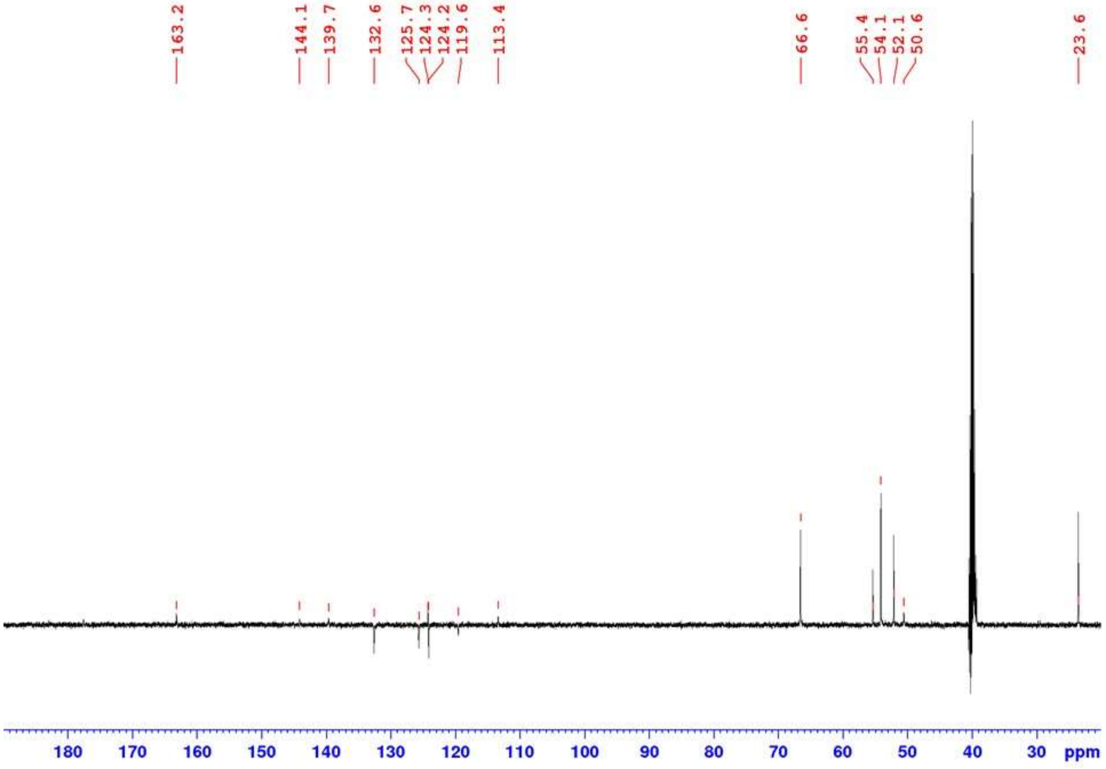

Supplement: Supplementary File 1 [file molecules-24-03502-s001.pdf]
